# Chitosan Use in Dentistry: A Systematic Review of Recent Clinical Studies

**DOI:** 10.3390/md17070417

**Published:** 2019-07-17

**Authors:** Marco Cicciù, Luca Fiorillo, Gabriele Cervino

**Affiliations:** 1 Department of Biomedical and Dental Sciences and Morphological and Functional Imaging, Messina University, Messina 98100, Italy; 2 Multidisciplinary Department of Medical-Surgical and Odontostomatological Specialties, University of Campania “Luigi Vanvitelli”, Naples 80100, Italy

**Keywords:** chitosan, dentistry, oral surgery, dental material, marine drugs

## Abstract

This study aims to highlight the latest marine-derived technologies in the biomedical field. The dental field, in particular, uses many marine-derived biomaterials, including chitosan. Chitosan that is used in different fields of medicine, is analyzed in this review with the aim of highlighting its uses and advantages in the dental field. A literature search was conducted in scientific search engines, using keywords in order to achieve the highest possible number of results. A review of randomized controlled trials (RCT) was conducted to evaluate and process all the relevant results for chitosan and oral health. After a screening and a careful analysis of the literature, there were only 12 results highlighted. Chitosan performs different functions and it is used in different fields of dentistry in a safe and effective way. Among the uses of chitosan, we report on the remineralizing property of chitosan which hardens tissues of the tooth, and therefore its role as a desensibilizer used in toothpastes. According to our systematic review, the use of chitosan has shown better surgical healing of post-extraction oral wounds. Furthermore, some studies show a reduction in bacterial biofilm when used in dental cements. In addition, it has antibacterial, antifungal, hemostatic and other systemic properties which aid its use for drug delivering.

## 1. Introduction

### 1.1. Rationale

In recent years, progress in the scientific and biomedical field has allowed the creation of new biomaterials that are increasingly efficient and safe. Often the process for obtaining biomaterials is difficult and expensive. Many steps have been made in the field of marine-based biomaterial research [1,2,3]. Currently, there are effective applications of marine-derived biomaterials in surgical, orthopedic, reconstructive plastic surgery, aesthetic, and dental material biomaterials [4,5]. Often the advantage of marine-derived biomaterials, such as collagen, is closely related to the quantity obtainable. One of the materials used in different fields of dentistry and which plays different roles is chitosan. Chitosan is a macromolecule formed by the repetition of D-glucosamine, which is derived from the deacetylation of chitin, obtained from the shells of marine crustaceans (in particular from crabs and prawns). It is a fiber, chemically similar to cellulose, and it is indigestible. Chitosan is a natural nontoxic, biocompatible, biodegradable polysaccharide with tested antibacterial properties [6]. The major properties of chitosan include biocompatibility, safety, biodegradability, antimicrobial activity, and the ability to form film and gel [7,8,9]. In conservative dentistry it has been used for the prevention of caries, as well as in to the field of conservative dentistry surgery [2].

### 1.2. Aims

The aim of this study is to systematically review the use of chitosan, and possibly the defects and merits of its use, in dentistry. A review of randomized controlled trials (RCT) was conducted to evaluate the use of chitosan in relation to other biomaterials or with different dosages, and therefore evaluate its potential.

## 2. Results

### 2.1. Manuscript Collection and Search Strategy

**Figure 1:**
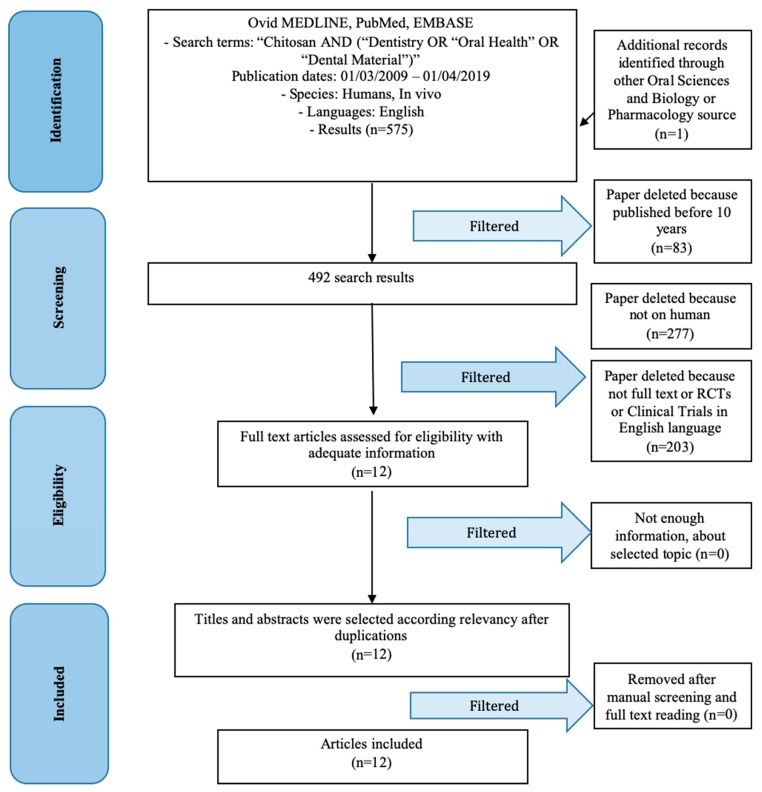
PRISMA (preferred reporting items for systematic reviews and meta-analyses) flow chart.

The first literature search produced 574 results. Subsequently, filters were applied to make the search and results more specific. First, we evaluated studies from the last 10 years (492 studies), studies on humans (215 studies), studies accessible in full-text (200, to be able to analyze the results), RCT, and then studies in the English language (3 studies excluded). Only 12 studies were included in this review (Figure 1). 

### 2.2. Study Characteristics

The results obtained were subsequently categorized into a chitosan field of use. The studies taken into consideration by this review are all randomized controlled trials (RCTs) and clinical trials. Each article presents information about the use of chitosan drugs in different fields of dentistry. The information has been related to each other and is summarized in Table 1. Zeza et al. [10] reported a better periodontal condition by using chitosan modified brushing. Pippi et al. [11] reported a significant reduction of healing time and bleeding time after dental extraction, however, pain reduction was not significant. Camacho-Alonso et al. [12] reported no significant differences in “bacterial colony forming unit” between the use of hypochlorite sodium or photodynamic therapy (PDT) and the use of PDT and chitosan. According to Mishra et al. [13] there was a significant reduction on biofilm formation and biomechanical characteristics of dental cements when chitosan was used in the mixture, as well as a better compressive strength with *P* < 0.001 statistical value. Atai et al. [14] reported no significant results on clinical signs to prevent mycosis (*P* > 0.05). Mandrazo-Jimenez et al. [15] reported no significant differences between swelling, pain, and wound healing after oral surgery maneuvers. Instead, Lopez-Lopez et al. [16] reported significant results for pain, inflammation, wound quality management, and analgesic usage after oral surgery with the use of chitosan. Mo et al. [17] reported no significant results with the use of a chitosan dressing for pain management and wound healing quality. Schlueter et al. [18,19] reported significant and no significant results in two different studies about tissue loss. Uysal et al. [20] reported a better condition against demineralization with the use of aloe and chitosan products. Mohire et al. [21] reported significant results using chitosan toothpaste against oral bacterial count.

### 2.3. Risk of Bias Within the Studies

It was not possible to carry out a univocal statistic between the articles as these evaluate the use of chitosan under different dental fields, however, the risk of bias was analyzed for each article individually and outlined in Table 1 and Table 2.

### 2.4. Risk of Bias Across the Studies

Numerous limitations have arisen from the present revision. Current analysis of the data extracted from studies written in English language only could introduce a publication bias. 

### 2.5. Evaluation of Studies

Statistical analysis of the studies was singularly analyzed. The studies seem to show useful information regarding the use of chitosan in dentistry and different dental fields are affected by the results of this review. Our multidisciplinary and skilled research team in different dental fields was able to examine all the data without external aids and draw important conclusions. A univocal statistic could not be made, because the materials used had chitosan but were different.

## 3. Discussion

The analysis of the results that will be conducted in this section certainly shows a variety of uses of chitosan in the dental field. Many different biomaterials of marine origin are used in the medical and dental field today [22,23,24,25,26,27,28]. In particular, marine biology has undergone an increase from a medical research point of view, thanks to the characteristics of the biomaterials that are developed and synthesized. Some additional authors, such as Min et al. [29] and Arnaud et al. [30] were considered (Table 3). Min et al. evaluated the use of chitosan-based hydrogels for bone morphogenetic protein-2 (BMP-2) delivery. According to authors, in view of the biocompatible and biodegradable properties of this chitosan gel, it can be used for bone regeneration and bone repair, based on the evaluation of a case of cancer-related osteosequestrectomy [31,32,33]. BMP are largely studied in dentistry and in oral surgery for bone regenerative techniques [34,35,36,37,38]. The use of BMP has also been promoted in the literature that reported good results [39,40]. According to Zeza et al. [10] a chitosan brushing could be a useful instrument for professional plaque removal. Chitosan brushing was shown to reduce clinical signs of per-implant inflammation, bleeding on probing, as well as stabilizing bone level. Pippi et al. [11] conducted RCT to investigate the use of chitosan-derived hemostatic for bleeding control and reported that this chitosan-derived dressing for post-extractive socket is a valid and safe alternative in the absence of surgical wound or lacerations. Camacho-Alonso et al. [12] evaluated the antibacterial efficacy of photodynamic therapy (PDT) and chitosan compared to sodium hypochlorite (NaOCL), only chitosan, or only PDT. The study was conducted with the use of a scanning electron microscope to determine the area of contamination and a colony-forming unit on plates was also performed. The combination of PDT and chitosan showed better results as compared with the control groups. Mishra et al. [13] used chitosan and chlorhexidine-cetrimide modified glass ionomer cements and concluded that chlorhexidine glass ionomer cements would be effective on bacteria growing associated to dental caries. This type of modified cement has improved physical properties as compared to a chitosan modified cement or a conventional cement. According to Atai et al. [14] chitosan is a valid alternative because it is antifungal, and with its biocompatibility it is a candidate for antifungal mouthwash. Mandrazo-Jimenez et al. [15] evaluated the effects of a topical gel containing chitosan, 0,2% chlorhexidine allantoin, and dexpanthenol on wound healing [41]. An improved wound healing was reported but it was not related to better postoperative comfort [42]. Lopez-Lopez et al. [16] evaluated the properties of the same gel of the last considered study (chlorhexidine, dexpanthenol, allantoin, chitosan gel) as compared with bicarbonate oral rinse. This RCT was conducted on 47 patients and the researchers found that the results for the gel patients group were better than the results for the bicarbonate rinse group with respect to controlling pain and inflammation after dental surgery [43,44,45]. Mo et al. conducted an open multicenter comparative RCT [17] and concluded that chitosan dressings could improve healing and wound re-epithelialization, as well as reduce pain levels. In addition, this chitosan dressing is clinically safe and effective on post-surgery wounds. Schueter et al. [18] evaluated the efficacy of chitosan enhanced Sn(2+)-containing toothpaste as an anti-erosive, abrasive agent and found that it was a good therapy option for patients with oral acid impacts or hypersensibility with a significant result. Schlueter et al. [19] conducted a study to evaluate the properties of a chitosan toothpaste and suggested that F/Sn/chitosan toothpaste could provide good protection for patients who consume acidic foodstuffs. On the basis of their findings it was possible to conclude that this toothpaste may be safer for patients undergoing dental whitening or other invasive treatments. Uysal et al. [20] proposed a study about demineralization properties of toothpaste containing chitosan. Chitosan-containing dentifrice may reduce decalcification of enamel in patients with a poor oral hygiene. In this case it was deducted that chitosan can prevent dentin hypersensibility and tooth decays. Arnaud et al. [30] conducted a chemical analysis and an optical coherence tomography to evaluate the de-remineralization effect of chitosan. This evaluation method has been used in dentistry, for dentin and enamel evaluating. Optical methods are useful in dentistry [46,47]. Finally, a 2010 RCT by Mohire and Yadav [21] was an interesting study that evaluated chitosan-based polyherbal toothpaste properties and reported that this toothpaste was a promising product as compared to standard oral hygiene products. Chitosan antimicrobial activity was proven, and it is a biocompatible and safer product. These properties, therefore, favors the hard tissues of the tooth, such as enamel and dentin [48], and allow chitosan to be a safe substance, which can be widely used without reported side effects. In fact, some toothpastes, despite having excellent properties reported in the literature, have many contraindications, especially for pediatric patients, for example, the presence of stannous fluoride [49,50,51]. The use of chitosan in surgery and periodontal/peri-implant should not be underestimated. Chitosan has an antibiotic and antifungal effect, and therefore it can prevent the onset of infectious processes in oral surgical wounds. At this point it would be useful to evaluate the effects on patients with osteonecrosis of the jaw, where wound superinfection is one of the most important aspects to manage [39,52]. The articles evaluated, therefore, are summarized based on results in the dental field where chitosan is used. These certainly concern two macro areas, conservative dentistry and oral surgery. For conservative dentistry, oral pathology, extractive surgery, periodontology and implant surgery have been considered and, for oral surgery, preventive dentistry, conservative dentistry, prosthesis, and endodontics have been considered as outlined in Table 1. The results for the field of oral surgery, and in particular in the management of healing timing, wound quality, and bleeding are positive [11,16]. Minor differences have been reported regarding pain management [11,16]. Furthermore, despite the bacteriostatic and mycostatic effect of chitosan, the articles do not report positive significant differences of chitosan use with respect to antifungals [14]. In the conservative field, the use of chitosan in cements improves their bacteriostatic characteristics, however, it does not present greater efficacy than topical disinfectants used in endodontics, such as hypochlorite [12]. This material is an excellent source for the development of dental biomaterials, benefiting from the many positive properties in the dental field. Although the literature provides clear results, it also includes the limitations of this compound. The theories regarding its positive characteristics, therefore, can be refuted because of the results of RCT. Furthermore, it would be useful to identify articles related to surgery, such as sutures, membranes or other biomaterials [53,54], enriched with chitosan. Chitosan has in fact presented excellent biocompatibility abilities, but it is not inert, it has a function in preventing infections of surgical wounds. Although they were not included in the review, as they were not part of the research parameters, certainly two other systemic properties deserve to be mentioned, which could be related to oral surgery and surgery in general. In fact, chitosan has other properties that can be exploited systemically. Chitosan has useful effects with respect to the maintenance of hypercholesterolemia [55], and it also has an important function in the management of arterial hypertension [56]. Indeed, its effects reported in blood are different, such as those already seen at the expense of hemostasis [11,57].

## 4. Materials and Methods 

### 4.1. Methods

#### 4.1.1. Protocol and Registration 

The methods of the performed revision and the inclusion criteria were specified in advance and documented in a protocol. The review was registered in the CRD York website PROSPERO, an international prospective register of systematic reviews. The protocol number is 134493 and the registration number is CRD.

The reporting of this systematic analysis adhered to the PRISMA statement.

#### 4.1.2. Focus Question

The following focus question was developed according to the population, intervention, comparison, and outcome (PICO) study design:

- What are the overall treatment outcomes of dental procedures using chitosan?

The following alternative focused question was also developed:

- Does chitosan use provide beneficial clinical outcomes?

#### 4.1.3. Information Sources

The search strategy incorporated examinations of electronic databases, supplemented by hand searches. A search of four electronic databases was carried out which included Ovid MEDLINE; PubMed; EMBASE; and a Dentistry and Oral Sciences Source consisting of biomaterials for relevant studies published in the English language from January 2009 to May 2019. 

A hand search was also performed in marine-derived materials and chitosan, including the MDPI publisher search engine. The search was limited to English language articles. A hand search of the reference lists in the articles retrieved was carried out to source additional relevant publications and to improve the sensitivity of the search.

#### 4.1.4. Search

The keywords used in the search of the selected electronic databases included the following: “Chitosan AND (Dentistry OR “Oral Health” OR “Dental Material”).

The choice of keywords was intended to collect and to record as much relevant data as possible without relying on electronic means alone to refine the search results.

#### 4.1.5. Selection of Studies

Two independent reviewers, from two different universities (Messina: G.C. and Naples: L.F.) singularly analyzed the obtaining papers in order to select the inclusion and exclusion criteria. Reviewers compared decisions and resolved differences by comparing the manuscripts and consulting with a senior reviewer, M. C. 

#### 4.1.6. Types of Selected Manuscripts

The review excluded studies involving animal and non-English language studies. Letters, editorials, case reports, and PhD theses were excluded. 

#### 4.1.7. Types of Studies

The review included all human RCTs and clinical trials published between January 2009 and May 2019, on the topic of biomaterial with chitosan used in dentistry.

#### 4.1.8. Disease Definition

The authors of this review classified the case definition of “chitosan use in dentistry” of each selected paper, if there was a documented use of chitosan in any of the different fields of dentistry.

#### 4.1.9. Inclusion and Exclusion Criteria

The full text of all studies of possible relevance was obtained for assessment against the following inclusion criteria:

- Chitosan description or use;
- Dental treatment using chitosan;
- RCTs or clinical trials.

The applied exclusion criteria for studies were as follows:

- Studies involving patients with specific diseases, immunologic disorders, uncontrolled diabetes mellitus, osteoporosis, or other implant risk related systemic conditions;
- Not enough information regarding the selected topic;
- Articles published prior to January 1, 2009;
- No access to the title and abstract in English language.

#### 4.1.10. Sequential Search Strategy

After the first literature analysis, all article titles were screened to exclude irrelevant publications, case reports, and the non-English language publications. Then, researches were not selected based on data obtained from screening the abstracts. The final stage of screening involved reading the full texts to confirm each study’s eligibility, based on the inclusion and exclusion criteria.

#### 4.1.11. Data Extraction

The data were independently extracted from studies in the form of variables, according to the aims and themes of the present review, as listed below.

#### 4.1.12. Data Collections

Data were collected from the included articles and arranged in the following fields:

“Author (Year)” revealed the author and year of publication;

“Type of study” indicated the type of the study;

“object of research” described the number of patients, animals or models examined;

“Field of use” described the dental field of chitosan use;

“Chitosan formulation or posology” described types chitosan used and control groups in different lines;

“Evaluation method” described the type of evaluating on sample;

“Risk of bias” indicated risk of bias information (not relevant for Table 3);

“Statistic” indicated Information about statistical results with respect to the evaluated method (Table 1 and Table 3).

#### 4.1.13. Risk of Bias Assessment

For the included studies, assessment of risk of bias was undertaken independently and in duplicate by the two authors, during the data extraction process. It was conducted using the Cochrane collaboration two-part tool used for assessing risk of bias.

The following possible sources of bias were addressed: random sequence generation (selection bias); allocation concealment (selection bias); blinding of participants and personnel (performance bias and detection bias); incomplete outcome data (attrition bias); selective reporting (reporting bias); and other bias (examiner blinding, examiner calibration, standardized follow-up description, standardized residual graft measurement, and standardized radiographic assessment). The authors’ judgment for each source of bias item was assigned for each study in the data extraction table (Table 1, Table 2 and Table 4). An overall risk of bias was then assigned to each trial according to Higgins et al. [58,59,60,61,62,63]. The levels of bias were classified as follows: low risk, if all the criteria were met; moderate risk, when only one criterion was missing; high risk, if two or more criteria were missing; and unclear risk, if too few details were available to make a judgement of certain risk assessment. In detail, a risk of bias evaluation includes a selection bias that is stated for adequate randomization, and to allocation concealment and baseline characteristics of an article. We should consider performance bias and unintended exposures or protocol variation, detection bias, with blinded or non-blinded subject, assessor blinded (with hard or soft outcomes), and measurement bias. Attrition bias is about incomplete outcome data, and reporting bias is about a selective outcomes reporting. All these parameters could take low risk, high risk, and unclear risk [64].

### 4.2. Chitosan

Chitosan, in dentistry, has been used in various studies to prevent tooth decay. Chitin is a cellulose-like biopolymer found mainly in the exoskeleton of marine animals such as shrimp, crabs, or lobsters. Chitin can also be found in mushrooms and yeasts. Chitosan is a chemically processed form of chitin. "Squid pens," waste shell by-products of squid processing, are a renewable and inexpensive source of chitosan. Furthermore, different formulations of chitosan are presented on the market characterized by different pH and different materials. There are gels based on chitosan containing lactic acid, some containing distilled water, and others containing chlorhexidine. The antibacterial activity of chitosan is strongly influenced by its formulation [65,66].

It has been associated with antibacterial effects on *Streptococcus mutans*, on *Actinomyces actinomycetemcomitans* and on *Porphyromonas gingivalis*. The bacteria contained in the plaque represent the first risk factor in the onset of primary and secondary caries, per-implant, and periodontal disease [67,68] or other systemic disease, such as neurodegenerative disease, by recent findings [69,70]. These species are able to penetrate into the microgaps that are created between the restorative material and the dental tissue. Therefore, by reducing the number of bacteria at the resin-tooth interface [47,71], the incidence of secondary caries may also be decreased. Therefore, the incorporation of antimicrobial agents in dental resin materials can be effective for the prevention of secondary caries. Although fluorine and chlorhexidine are the most commonly incorporated antimicrobial agents in resinous materials, their release does not continue for a long time. Furthermore, the mechanical properties of resinous materials change and significantly reduce their adhesion strength [72]. Currently, the research aims to increase the durability of the resin-dentin bond, in other cases between resinous materials and other dental cements [47]. In some studies, it has been hypothesized that chitosan, modified with methacrylic groups, is able to covalently bind to the resin of dental restorations and, because of the presence of residual positive charges on the polysaccharide, to interact electrostatically with the demineralized dentin. Therefore, by introducing chitosan methacrylate in the primer of a three-step "etch and rinse" adhesive system we can achieve good adhesion values and good stability of the hybrid layer when subjected to mechanical simulation of chewing and thermal stress. It would also seem to improve the characteristics of mucus-adhesion to the enamel, producing a better remineralization. Unlike other tissues of the human body, enamel and dentin alone do not undergo repair because there are no cells inside them that can be activated to begin a repair process.

Over time, numerous products based on phosphate, calcium, and, above all, fluorine have been developed, which may have some effect in combating demineralization by interacting with damaged structures and stopping their dissolution. However, these are solutions limited in time, while the ideal would be to be able to produce a regeneration of the tissues, able to lead to the recovery of the full functionality of the tooth [71,73]. The maximum effect of this material is on gram-positive bacteria such as *Streptococcus sanguis*, *S. mutans*, *Streptococco mitus*, *Streptococcus salivarius*, and yeasts [74]. It has some other favorable characteristics and applications such as the prevention of demineralization, prevention of plaque and biofilm formation, stimulation of salivary secretion, antitumor activity, haemostatic properties, improvement of wound recovery, antihypertensive properties, reduction of serum cholesterol, delivery system drug, implant lining, bone tissue engineering and bone regeneration, blood vessel repair, and nerve repair. Under physiological and biological conditions, this material does not stimulate the immune system.

Some studies have also shown that vitamin C, that is ascorbic acid, helps activate the chitosan taken in the stomach and intestine, thus forming this lipid-absorbing gel. Therefore, it helps to reduce the absorption of cholesterol and fats present in food, and therefore it is proposed for obesity, Crohn’s disease and treatment for the complications of dialysis (including hypercholesterolemia) [75]. Moreover, it is important to take into account that, unlike traditional cholesterol-lowering drugs, known for their side effects such as hair loss, itching, and sleep problems, chitosan is much more tolerated. It can also be used to stimulate the properties of insulin. It has in fact been noted that chitosan supports insulin by helping to reduce blood glucose levels [76]. However, in some circumstances it may have contraindications such as interference with the anticoagulant warfarin and interference with patients presenting with bleeding disorders or haemostasis [57,77]. Chitosan supplements taken by mouth are considered safe even if taken for six consecutive weeks, but information on their safety during pregnancy is lacking. Furthermore, during the treatment, side effects may appear, such as mild stomach discomfort, constipation or meteorism, and allergic reactions in people allergic to crustaceans, even if the allergy to these foods depends on proteins not present in the exoskeleton from which this polysaccharide is extracted. It is easily absorbed by the human body, reacts with body fluids, and its physical and chemical properties are easily adjustable. Certainly, by improving the oral health conditions of patients’ mouths, reducing the bacterial load, and reducing the state of inflammation, it is also possible to improve some systemic parameters, such as glycaemia [76], and also their psychological conditions. Different studies evaluate the influence of oral health on patients’ psychological conditions [77,78,79,80], using tests such as the Oral Health Quality of Life Related (OHQoLR). The psychological conditions of patients in turn further influence the immune system [81,82,83]. The use of these marine-derived materials, with properties that we have seen in this study, is certainly supportive of oral health and the maintenance of dental therapies.

## 5. Conclusions

The results analyzed in this study show that chitosan is a safe compound to use, with many positive properties for applications in oral surgery and restorative dentistry. Chitosan is used in these different fields, with good results and no reported side effects. Unfortunately, the advantageous of its use were not always statistically significant. As we have seen during this review, for example, using this compound, linked to synthetic dental materials could improve its characteristics from a bacteriostatic or mycostatic point of view. In recent years, bioengineering has been moving towards the creation of new biomaterials, which are active and have beneficial properties with respect to therapies. At the same time, the marine-derived materials are protagonists of growing scientific and clinical interest which is also guaranteed by their availability.

## Figures and Tables

**Table 1:** Synthesis of data according to data collections paragraph.

Author (Year)	Type of Study	Object of Research	Field of Use	Chitosan Formulation or Posology	Evaluation Method	Risk of Bias	Statistic
Zeza et al. (2017) [10]	Clinical trial	15 patients	Periodontology, implantology	Chitosan brush	Modified plaque index, modified bleeding on probing, probing depth	Moderate	Significant
Pippi et al. (2017) [11]	RCT	20 patients	Oral surgery	HemCon^®^ dental dressing	Time, bleeding time, pain	Low	Significant (*P* = 0.00452, *P* = 0.0278) Not significant (*P* = 0.3843)
				Common hemostatic sponge CollaPlug, Zimmer Dental^®^			
Mishra et al. (2017) [13]	RCT	50 patients (pediatric)	Dental material, Restorative dentistry	Glass ionomer cement Ketac 3M^®^	Biofilm evaluation, agar diffusion test, compressive strength and flexural strength evaluation	low	Significant (Lower biofilm to group II and III *P* < 0.001, compressive strength lower in group III, similar flexure strength through groups)
				Chlorhexidine into glass ionomer cement			
				Chlorhexidine-chitosan mixture into glass ionomer cement			
Atai et al. (2017) [14]	RCT	40 patients	Oral pathology	Chitosan solution 1%wt pH 5	Clinical signs (erytematosis and pain), mycelia and blastospores count	low	Not significant (*P* > 0.05, *P* > 0.05)
				Nystatin oral drops 100,000 U/mL			
Mandrazo-Jimenez et al. (2016) [15]	RCT	50 patients	Oral surgery	topical gel composed of chitosan, 0.2% chlorhexidine, allantoin, and dexpanthenol	Swelling, pain, wound healing appearance	low	Not significant
				Any gel			
Lopez-Lopez et al. (2015) [16]	RCT	47 patients	Oral surgery	topical gel composed of chitosan, 0.2% chlorhexidine, allantoin and dexpanthenol	Pain, inflammation, analgesic pill usage, cicatrization quality	Low	Significant (*P* = 0.0001, *P* = 0.0001, *P* < 0.05, *P* = 0.0001)
				Bicarbonate rinse			
Mo et al. (2015) [17]	RCT	90 patients	Oral surgery	Chitosan wound dressing 10 × 10 cm	Wound area reduction, pain, wound depth, exudate	low	Not significant
				Control group			
Schlueter et al. (2014) [18]	RCT	10 patients with appliances of human enamel specimens	Restorative dentistry	F/Sn = 1400 ppm F(−), 3500 ppm Sn(2+)	Tissue loss measurement	Moderate	Not significant
				F/Sn/chitosan = 1400 ppm F(−), 3500 ppm Sn(2+), 0.5% chitosan			
				Placebo toothpaste			
Schlueter et al. (2013) [19]	RCT	27 patients	Restorative Dentistry	F/Sn = 1400 ppm F(−), 3500 ppm Sn(2+)	Tissue loss measurement	Moderate	Significant
				F/Sn/chitosan = 1400 ppm F(−), 3500 ppm Sn(2+), 0.5% chitosan			
				Placebo toothpaste			
Uysal et al. (2011) [20]		16 patients	Orthodontics, Restorative dentistry	Aloe Dent (with chitosan)	Demineralization around orthodontic brackets	Moderate	Significant
				Sensodyne Mint			
Mohire et al. (2010) [21]	Clinical trial	/	Restorative dentistry	Polyherbal toothpaste with chitosan	Clinical evaluation, oral bacterial count	Moderate	Significant
				Chlorhexidine mouthwash			
				Placebo			

**Table 2:** Risk of bias results evaluation.

Author (Year)	Risk of Bias			
	Unclear	Low	Moderate	High
Zeza et al. (2017) [10]			√	
Pippi et al. (2017) [11]		√		
Camacho-Alonso et al. (2017) [12]		√		
Mishra et al. (2017) [13]		√		
Atai et al. (2017) [14]		√		
Mandrazo-Jimenez et al (2016) [15]		√		
Lopez-Lopez et al. (2015) [16]		√		
Mo et al. (2015) [17]		√		
Schlueter et al. (2014) [18]			√	
Schlueter et al. (2013) [19]			√	
Uysal et al. (2011) [20]			√	
Mohire et al. (2010) [21]			√	

**Table 3:** Additional results supporting use of chitosan (in vitro studies).

Author (Year)	Type of Study	Object of Research	Field of Use	Chitosan Formulation or Posology	Evaluation Method	Statistic
Camacho-Alonso et al. (2017) [12]	RCT	100 teeth on patients	Endodontics	2.5% NaOCl	Colony-forming unit, SEM analysis	Low (not significant)
				PDT		
				Chitosan 3 mg/mL		
				PDT + chitosan 3 mg/mL		
				Positive control		
				Negative control		
Min et al. (2019) [29]	Article	\	Oral surgery; Periodontology	Chitosan Aladdin Inc (Shanghai, China)^®^	In vitro release of BMP-2	Significant
Arnaud et al. (2010) [30]	In vitro study (Random experimental design)	Tooth layers	Restorative dentistry	Chitosan Aldrich Corporation as chitosan crustacean Sigma^®^	Optical coherence tomography measurements	Significant

**Table 4:** Journal results impact factor list.

Author (Year)	Journal Last Impact Factor
Zeza et al. (2017) [10]	Emerging
Pippi et al. (2017) [11]	2.164
Camacho-Alonso et al. (2017) [12]	1.620
Mishra et al. (2017) [13]	0.53
Atai et al. (2017) [14]	0.853
Mandrazo-Jimenez et al (2016) [15]	1.671
Lopez-Lopez et al. (2015) [16]	2.665
Mo et al. (2015) [17]	2.69
Schlueter et al. (2014) [18]	2.386
Schlueter et al. (2013) [19]	2.188
Uysal et al. (2011) [20]	0.59
Mohire et al. (2010) [21]	0.37
